# Response Characteristics and Community Assembly Mechanisms of nirS-Type Denitrifiers in the Alpine Wetland under Simulated Precipitation Conditions

**DOI:** 10.3390/biology13080596

**Published:** 2024-08-07

**Authors:** Ni Zhang, Kelong Chen, Ji Chen, Wei Ji, Ziwei Yang, Zhirong Chen

**Affiliations:** 1 Qinghai Province Key Laboratory of Physical Geography and Environmental Process, College of Geographical Science, Qinghai Normal University, Xining 810008, China; zhangni0224@163.com (N.Z.); jiwei100500@163.com (W.J.); 15756789182@163.com (Z.Y.); 2 Key Laboratory of Tibetan Plateau Land Surface Processes and Ecological Conservation (Ministry of Education), Qinghai Normal University, Xining 810008, China; 3 National Positioning Observation and Research Station of Qinghai Lake Wetland Ecosystem in Qinghai, National Forestry and Grassland Administration, Haibei 812300, China; 4 State Key Laboratory of Loess and Quaternary Geology, Institute of Earth Environment, Chinese Academy of Sciences, Xi’an 710061, China; chenji@ieecas.cn; 5 Department of Earth and Environmental Science, Institute of Global Environmental Change, School of Human Settlements and Civil Engineering, Xi’an Jiaotong University, Xi’an 710061, China; 6 Guanzhong Plain Ecological Environment Change and Comprehensive Treatment National Observation and Research Station, Xi’an 710061, China; 7 College of Resources, Environment and Life Sciences, Ningxia Normal University, Guyuan 756099, China; 82022008@nxnu.edu.cn

**Keywords:** Qinghai-Tibet Plateau, climate change, extreme precipitation, characteristics of microbial communities, nitrogen cycle

## Abstract

**Simple Summary:**

The nitrogen cycling process in alpine wetlands is profoundly affected by precipitation changes. Utilizing high-throughput sequencing analysis of nirS-type functional genes, this study delved into the dynamic response mechanism of nirS-type denitrifiers to precipitation changes in the alpine wetland of Qinghai Lake. The findings revealed that a 50% increase in rainfall shifted the community assembly process of denitrifiers from deterministic to stochastic. Dominant microflora at the genus level responded significantly to precipitation changes, with aer-obic bacteria comprising the majority of differentially abundant taxa. As precipitation in-creased, the complexity of the microbial interaction network decreased. Precipitation notably emerged as the primary regulator of nirS-type denitrifiers, accounting for 51% of the variation in community composition. In summary, this study offers a fresh perspective for investigating the ecological processes of nitrogen cycling in alpine ecosystems.

**Abstract:**

The nitrogen cycling process in alpine wetlands is profoundly affected by precipitation changes, yet the dynamic response mechanism of denitrifiers to long-term precipitation shifts in the alpine wetland of the Qinghai-Tibet Plateau remains enigmatic. Utilizing high-throughput sequencing analysis of nirS-type functional genes, this study delved into the dynamic response mechanism of nirS-type denitrifiers to precipitation changes in the alpine wetland of Qinghai Lake. The findings revealed that nirS-type denitrifiers in the alpine wetland of Qinghai Lake were primarily Proteobacteria, and Alpha diversity exhibited a negative correlation with the precipitation gradient, with deterministic processes predominating in the community assembly of denitrifying microbes. A 50% increase in rainfall shifted the community assembly process of denitrifiers from deterministic to stochastic. Dominant microflora at the genus level responded significantly to precipitation changes, with aerobic bacteria comprising the majority of differentially abundant taxa (55.56%). As precipitation increased, the complexity of the microbial interaction network decreased, and a 25% reduction in precipitation notably elevated the relative abundance of three key functional groups: chemoheterotrophic, aerobic chemoheterotrophic, and nitrogen fixation. Precipitation notably emerged as the primary regulator of nirS-type denitrifiers in the alpine wetland of Qinghai Lake, accounting for 51% of the variation in community composition. In summary, this study offers a fresh perspective for investigating the ecological processes of nitrogen cycling in alpine ecosystems by examining the diversity and community composition of nirS-type denitrifiers in response to precipitation changes.

## 1. Introduction

Nitrous oxide (N_2_O) is a potent greenhouse gas with a radiative effect more pronounced than carbon dioxide, significantly contributing to global warming since the Industrial Revolution [1,2,3]. Precipitation, the most crucial ecological factor shaping biological communities, largely determines spatiotemporal variability of soil moisture and its dynamic changes, significantly influencing soil N_2_O emissions [4]. Previous research has yielded varied results, potentially due to nonlinear ecosystem responses to precipitation variability [5]. Zhang et al. found in their study of high-altitude meadows that dramatic changes in precipitation led to a significant increase in N_2_O flux in the alpine meadows of Zoige compared to natural conditions [6]. Zhao et al. investigated the risks of greenhouse gas emissions and major influencing factors in northeastern agricultural ecosystems, revealing that these emissions are influenced by precipitation events, resulting in increased postrainfall N_2_O emissions [7]. Zhang et al. also examined dynamic N transport and N_2_O emissions during rainfall events in coastal rivers, demonstrating that N_2_O emissions rise with rainfall, particularly during intense rainfall events [8]. Hu et al. explored the effects of warming and reduced precipitation on soil respiration and N_2_O flux, highlighting a significant reduction in cumulative N_2_O emissions with decreased precipitation [9]. In contrast, Zhang et al. studied how reduced precipitation affects field soil N_2_O emissions, finding that moderate reductions enhance nitrogen cycling and soil nitrous oxide emissions in plateau grasslands [10]. Additionally, Ni et al. discovered that short-term changes in precipitation intensity can stimulate CO_2_ emissions but do not affect N_2_O flux [11].

Denitrification, primarily driven by micro-organisms, is a key process contributing to N_2_O emissions. Changes in microbial community characteristics have a sustained impact on soil N_2_O emissions [12,13,14,15]. Recent research further underscores that microbial denitrification processes lead to increased soil N_2_O emissions [16]. The catalytic reaction at the heart of denitrification is mediated by nitrite reductase (nir), facilitated by structurally distinct but functionally similar nirS and nirK genes [17,18]. While both nirS and nirK enzymes are functionally and physiologically equivalent, nirS exhibits a broader environmental distribution compared to nirK [19,20]. Moreover, nirS-type denitrifying bacteria contribute more significantly to N_2_O emissions than nirK-type bacteria [21]. Research by Mosier and Francis further supports a close correlation between N_2_O emissions and nirS-type bacterial communities [22]. Alpine wetlands, due to their regional specificity, are profoundly influenced by variations in precipitation, which affect their ecological functions and nutrient cycles. Since 1960, there has been an increase in precipitation variability in alpine wetlands, a trend expected to continue due to climate change [23,24]. The Qinghai-Tibet Plateau features extensive alpine wetlands covering approximately 5.1 × 10^3^ km^2^ [25,26]. Recent precipitation events in this region have shown a trend towards less frequent but more intense occurrences [27]. This climate trend is projected to intensify further [28,29], significantly impacting the soil denitrification process in alpine wetlands.

The Qinghai Lake basin, situated in the northeastern part of the Qinghai-Tibet Plateau, serves as a crucial barrier for maintaining ecological security within the region [30]. It is also a national-level nature reserve recognized for its internationally significant wetlands [31]. However, the response mechanisms of denitrifiers in the Qinghai Lake basin to changes in precipitation remain poorly understood. This study utilized high-throughput sequencing technology to detect nirS genes in the river-source wetlands of Qinghai Lake, analyzing the impacts of long-term precipitation changes and environmental factors on the community of denitrifiers. Based on previous findings, we hypothesize that (1) the species richness and diversity of nirS-type denitrifiers are positively correlated with increased precipitation; (2) the complexity and stability of nirS-type denitrifiers are influenced by varying degrees of precipitation; and (3) increased precipitation may enhance the relative abundance of nirS-type denitrifiers due to the creation of anaerobic conditions. In summary, the results of this study enrich the research on functional processes within the high-altitude wetland ecosystems of the Qinghai-Tibet Plateau and provide a scientific basis for studying the microbial mechanisms involved in nitrogen cycling within the biogeochemical cycles of high-altitude wetlands.

## 2. Materials and Methods

### 2.1. Overview of the Study Area

**Figure 1:**
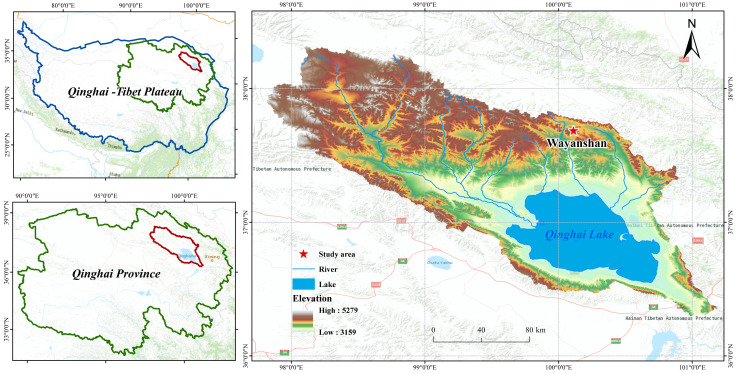
Sampling point distribution.

The Tibetan Plateau, as one of the sensitive regions to global climate change, exhibits distinct warming and wetting characteristics. Notably, precipitation has been increasing at a rate of 2.2% per decade, significantly impacting typical wetland ecosystems such as Qinghai Lake. The study area was established in 2018 at the Wayanshan Experimental Station (37°43′~37°46′ N, 100°01′~100°05′ E) at the head of the Shaliu River in Qinghai Lake. The distribution of the sample sites is shown in Figure 1. The elevation of the area is approximately 4517 m above sea level. Using natural precipitation as the control, field experiments with precipitation variations of ±25% and ±50% were conducted. The reduction of natural precipitation was achieved by installing equidistant inclined diversion channels in different areas. Rainwater collected in the diversion trough was directed into a horizontal tank in the convergence trough, and precipitation was increased using a spray device. Samples were classified as follows: Wck (natural control), ZA (50% increase in rain), ZB (25% increase in rain), JA (50% decrease in rain), and JB (25% decrease in rain). The altitude of the study site ranges between 3720 and 3850 m, with a notable diurnal temperature range and stark contrasts. The annual average temperature is −3.31 °C, and the annual average precipitation is 420 mm, characteristic of a typical plateau continental climate.

### 2.2. Sample Collection and Physicochemical Determination

Soil samples were collected in June 2022. Each sample consisted of five cores that were collected at 0–10 cm of topsoil using a five-point sampling method using a soil drill (4.5 cm diameter). A total of 15 soil samples were collected, i.e., 5 treatments × 3 replicates. The collected soil samples were screened (pore size was 2 mm), large particles, roots, and rocks were removed, stored in ice packs, and immediately returned to the laboratory. The samples were stored at −4 °C for physicochemical analysis and stored at −80 °C for molecular analysis.

The TDR-300 Soil Moisture Probe (Spectrum Technologies Inc., Plainfield, IL, USA) monitored soil moisture content. LI-8100 (LI-COR Inc., Lincoln, NE, USA) monitored soil temperature. Soil pH was determined using a pH probe (FE20-FiveEasy pH, Mettler Toledo, Germany) with a soil–water ratio of 1:2.5. The contents of total carbon (TC) and total nitrogen (TN) were determined using the Elemental Analysis System GmbH (Vario EL III, Frankfurt, Germany).

### 2.3. DNA Extraction and Polymerase Chain Reaction

Soil microbial DNA was extracted from 0.5 g of fresh soil, Specific primers Cd3aF (5′-GTSAACGTSAAGGARACSGG-3′) and R3cd (5′-GASTTCGGRTGSGTCTTGA-3′) were used to amplify nirS-type denitrifiers. Paired end sequencing of the PCR amplicon was performed on the Illumina MiSeq sequencer (Illumina, San Diego, CA, USA). Primer sequences were identified and removed by Cutadapt (v.1.9.1), spliced and filtered by Usearch (v.10.0) [32]. The DADA2 method in QIIME2 (v.2020.6) performed noise reduction and chimera sequence removal to obtain valid data and finally generated ASV [33]. Data annotation was performed using the NCBI nonredundant protein sequences (NR) database as the reference database.

### 2.4. Statistical Analysis

The MicrobiotaProcess package of R software (version 4.3.2) calculated the Alpha diversity index of microorganisms, the UpSetR package (version 1.4.0) mapped the ASV distribution. The PCAtools package (version 2.14.0) calculated the Beta diversity index, and the vegan package (version 2.6.6.1) performed nonmetric multidimensional scaling (NMDS), and Adonis and Anisom analyses based on the Bray–Curtis distance verified the grouping. Meanwhile, redundancy analysis from the vegan package (version 2.6.6.1) was based on linear functions. The psych package (version 2.4.3) computed correlations between the data, and the stats package’s aov function calculated *p*-values. Functional groups of denitrifying microorganisms were predicted using functional annotation of prokaryotic taxa (FAPROTAX) [34]. The linkET package (version 0.0.7.4) calculated correlations, the qcorrplot function plotted the correlation network heatmap, and the pheatmap package (version 1.0.12) generated the correlation heatmap. The piecewiseSEM package (version 2.3.0) was used to calculate and visualize structural equation models (SEM), enabling the assessment of both direct and indirect impacts of environmental variables on Alpha diversity and community composition of nirS-type denitrifiers. The betaNTI index was computed using the Picante package (version 1.8.2) based on null models, and the Raup–Crick (RCbray) index was determined using the microeco package (version 1.7.1) to assess the relative importance of deterministic and stochastic processes in the community assembly. The site map was created using ArcGIS (version 10.8).

## 3. Results

### 3.1. Response of Community Diversity of nirS-Type Denitrifiers to Precipitation Change

**Figure 2:**
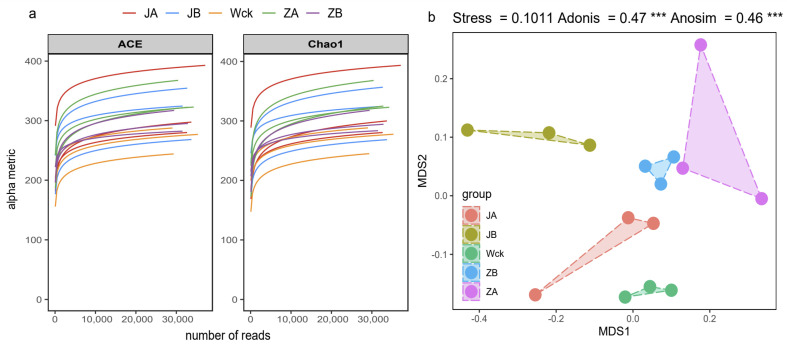
nirS sequencing results after four years of simulated precipitation treatment; (**a**) sample dilution curve; (**b**) analysis of differences between groups. Wck: natural contrast, ZA: 50% increase in rainfall; ZB: 25% increase in rainfall; JA: 50% decrease in rainfall; JB: 25% decrease in rainfall, and *** indicates *p* < 0.001.

**Figure 3:**
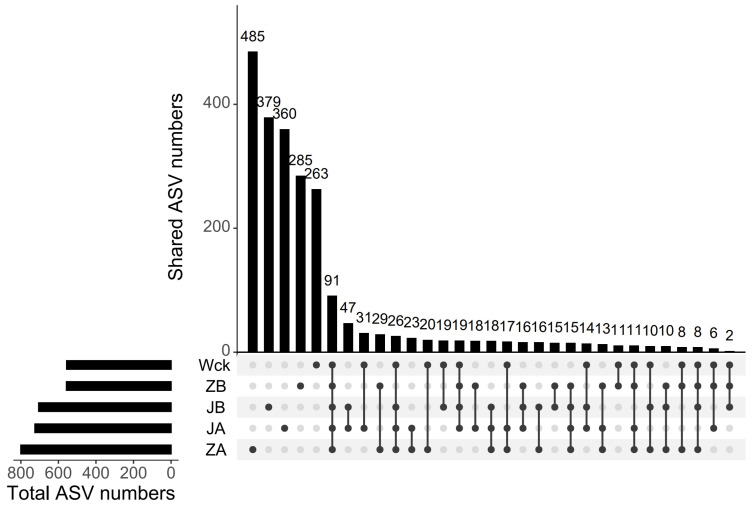
ASV distribution map of simulated precipitation treatment.

The sequencing curve of each sample tends to flatten out, indicating that the amount of data is sufficient to reflect the sample situation (Figure 2a). The sequence numbers of nirS-type denitrifiers in 15 samples ranged from 328,395 to 418,950. The sequences were clustered into ASVs using the DADA2 method, resulting in a total of 91 ASVs in the group. The unique counts of ASVs in JA, JB, Wck, ZB, and ZA were 360, 379, 263, 285, and 485, respectively (Figure 3). The Alpha diversity of the nirS-type denitrifiers was less affected by precipitation changes (Figure S1a). Compared to 50% and 25% rainfall reduction treatments, the 25% rainfall reduction and 25% rainfall increase treatments could enhance the richness of nirS-type denitrifiers. Additionally, the Shannon index and Simpson index of nirS-type denitrifiers were elevated with the rain reduction treatment (Figure S1a). Principal component analysis (PCA) revealed significant sample heterogeneity between groups under JA, JB, and ZA treatments (Figure S1b). Nonmetric multidimensional scaling (NMDS), Adonis, and Anosim were utilized to assess the validity of the groups, revealing significant variations in the nirS-type denitrifiers with changes in precipitation (*p* < 0.05; Figure 2b).

### 3.2. Response of Community Structure of nirS-Type Denitrifiers to Precipitation Change

**Figure 4:**
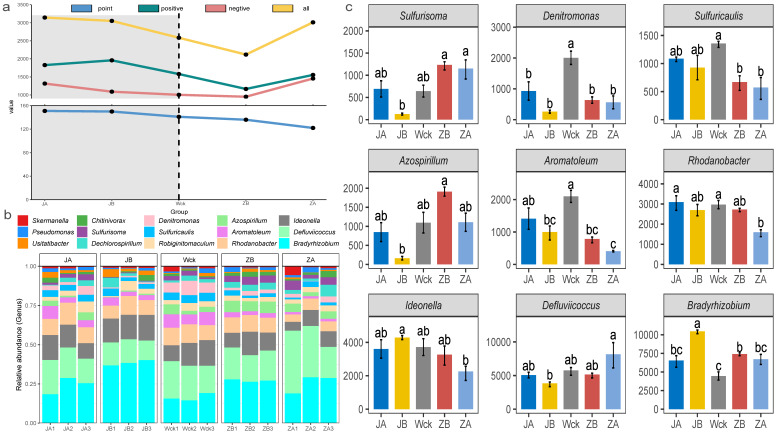
Microbial community composition of nirS-type denitrifiers after four years of simulated precipitation treatment; (**a**) community interaction network relationship analysis; (**b**) the community structure of genus level; (**c**) different flora at genus level; abc indicates significance, the same letter indicates no significant difference between groups (*p* > 0.05), and different letters indicate a significant difference between groups (*p* < 0.05).

The analysis of interaction network relationships among nirS-type denitrifiers in each group (Figure 4a) demonstrated that changes in precipitation have a significant impact on the total number of nodes and connections within microbial interaction networks. The microorganisms exhibiting significant interrelationships gradually decreased as precipitation increased, while the complexity of the microbial interaction network exhibited a trend of first decreasing and then increasing with increasing precipitation (Figure 4a). Further analysis of the community composition of nirS-type denitrifiers (Figure 4b) revealed that Proteobacteria (98.90%) were the dominant bacterial group in the alpine wetland. There were 14 dominant bacterial groups at the genus level, with a relative abundance greater than 1%, and *Bradyrhizobium* exhibited the highest relative abundance (24.51%). The relative abundance of microflora in nine genera was significantly affected by changes in precipitation, and the response characteristics of different microflora varied (Figure 4c). The relative abundance of *Bradyrhizobium* and *Ideonella* was the highest when precipitation decreased by 25%, whereas the relative abundance of *Defluviicoccus* peaked when precipitation increased by 50%. The response trend of *Azospirillum* and *Sulfurisoma* to changes in precipitation was consistent; their relative abundance was lowest when rainfall decreased by 25% and highest when rainfall increased by 25%. Additionally, *Rhodanobacter*, *Aromatoleum*, *Denitromonas*, and *Sulfuricaulis* exhibited a similar response trend to changes in precipitation. Their relative abundance was highest in natural precipitation, with *Denitromonas* being the most sensitive to moisture changes.

### 3.3. Response of Functional Groups of nirS-Type Denitrifiers to Precipitation Changes

**Figure 5:**
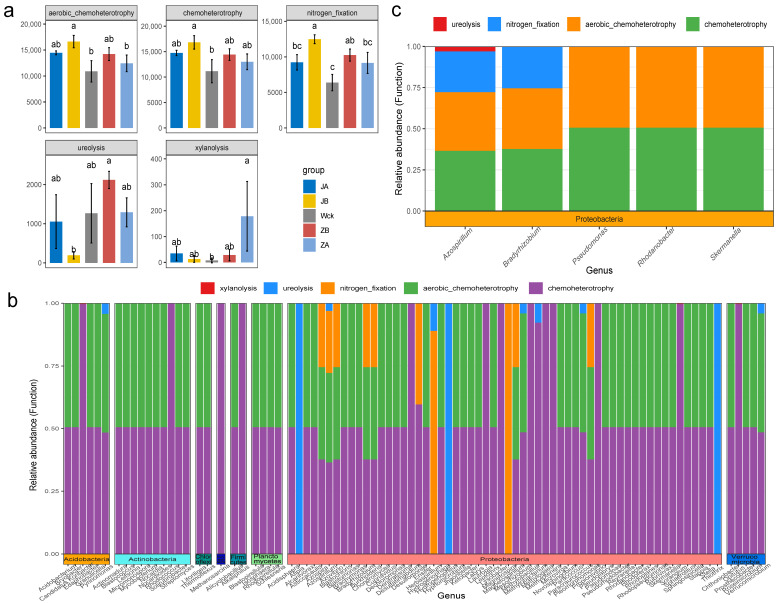
Functional groups of nirS-type denitrifiers after four years of simulated precipitation treatment; (**a**) different functional groups between groups; (**b**) microbial flora corresponding to differential functional groups; (**c**) the different functional groups correspond to the main genus level flora; abc indicates significance, the same letter indicates no significant difference between groups (*p* > 0.05), and different letters indicate a significant difference between groups (*p* < 0.05).

FAPROTAX predicted the functional groups of denitrifying microbial communities in the alpine wetland and identified 23 functional groups of nirS-type denitrifiers (Figure S1c). The functions of these microorganisms primarily focused on chemoheterotrophy (32.28%), aerobic chemoheterotrophy (31.59%), and nitrogen fixation (21.73%), accounting for over 85% of the total sequences. The results of statistical analysis indicated that the response trend of these three major functional groups to changes in precipitation tended to be consistent. Specifically, a 25% reduction in rainfall treatment significantly increased their relative abundance (*p* < 0.05) (Figure 5a). Ureolysis and xylanolysis also responded significantly to precipitation changes (Figure 5a). The relative abundance of ureolysis was highest when rainfall increased by 25%, while the relative abundance of xylanolysis was highest when rainfall increased by 50%. Reverse mapping revealed that these functional groups were distributed across 88 bacterial genera, belonging to 8 different phyla, with the majority being affiliated with the Proteobacteria phylum (Figure 5b). Further screening of major microflora at the genus level (with a relative abundance > 1%) (Figure 5c) revealed that five genera within the Proteobacteria phylum possessed chemoheterotrophic and aerobic chemoheterotrophic functions. Specifically, *Bradyrhizobium* and *Azospirillum* exhibited nitrogen fixation capabilities. Furthermore, *Azospirillum* also functioned in ureolysis.

### 3.4. Relationship between nirS-Type Denitrifiers and Environmental Factors

**Figure 6:**
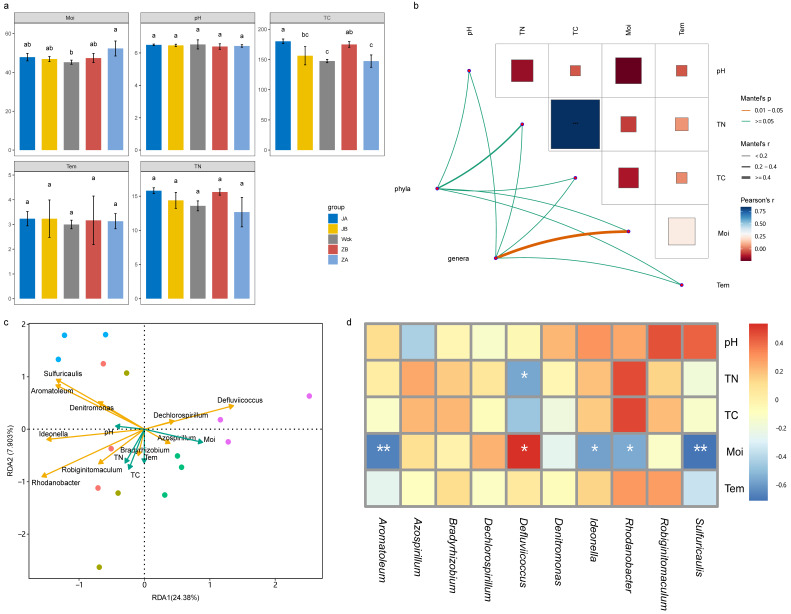
Physicochemical properties and correlation analysis of soil samples after four years of simulated precipitation treatment; (**a**) the content changes of physicochemical factors in different precipitation gradients; (**b**) network diagram of correlation between microbial community structure and physicochemical factors; (**c**) the redundancy analysis of genus level flora and physicochemical factor; (**d**) heatmap of correlation between genus level flora and physicochemical factors. Tem: soil temperature, Moi: soil moisture, TN: total nitrogen, TC: total carbon, pH: soil pH; abc indicates significance, the same letter indicates no significant difference between groups (*p* > 0.05), and different letters indicate a significant difference between groups (*p* < 0.05); * indicates *p* < 0.05, ** indicates *p* < 0.01, and *** indicates *p* < 0.001.

The results of statistical analysis indicated that pH, temperature (Tem), and total nitrogen (TN) did not exhibit significant responses to changes in precipitation, whereas moisture (Moi) and total carbon (TC) contents underwent notable alterations (Figure 6a). A 50% reduction in rainfall and a 25% increase in rainfall both resulted in a significant increase in TC content. Additionally, a 50% increase in precipitation led to a significant rise in soil moisture content. However, precipitation treatments at the remaining gradients had minimal effects on Moi and TC (Figure 6a). The correlation network diagram revealed a highly significant positive correlation between TN and TC (Figure 6b). This correlation was further supported by the consistent response trends observed for both parameters in response to precipitation changes. The community structure of nirS-type denitrifiers at the phylum level was not significantly influenced by environmental factors, whereas at the genus level, it was notably impacted by soil Moi content (Figure 6b). Redundancy analysis (RDA) also identified Moi as the most significant influencing factor of the nirS-type denitrifiers (Figure 6c). The correlation heatmap provided further clarification on the influence of physicochemical factors on the dominant strains of nirS-type denitrifiers (Figure 6d). Specifically, it was discovered that TN exhibited a significant negative correlation with *Defluviicoccus*, whereas Moi demonstrated a significant positive correlation with *Defluviicoccus*. On the other hand, Moi was negatively correlated with *Sulfuricaulis*, *Rhodanobacter*, *Ideonella*, and *Aromatoleum*.

**Figure 7:**
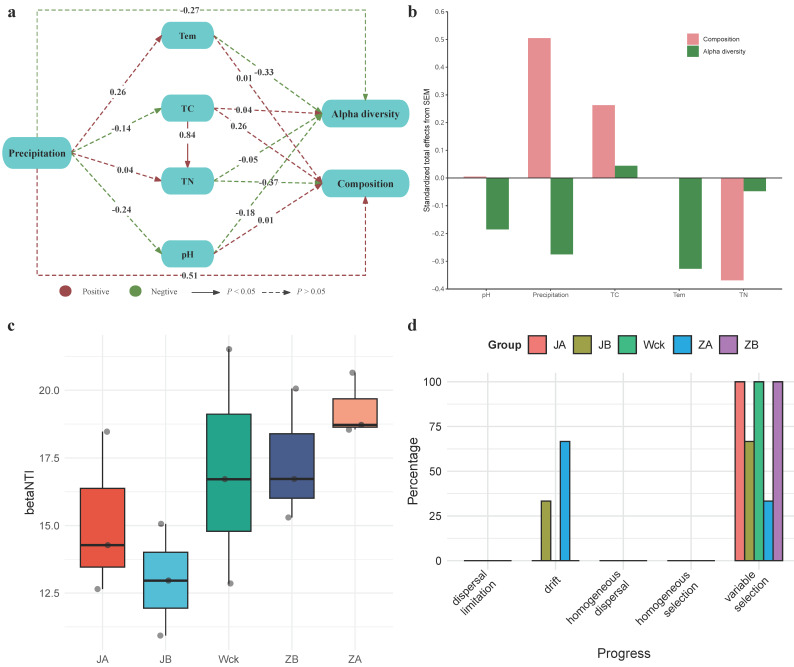
Influencing factors and community construction of nirS-type denitrifiers under precipitation treatment; (**a**) structural equation model (SEM); (**b**) overall standardized effects of each variable on microbial diversity index and community composition; (**c**) betaNTI index of different groups; (**d**) distribution of community construction process in different groups. Tem: soil temperature, TN: total nitrogen, TC: total carbon, pH: soil pH.

Structural equation modeling (SEM) was utilized to explore the direct and indirect roles of various variables in the Alpha diversity and community composition of nirS-type denitrifiers (Figure 7a,b). Specifically, precipitation had a more profound effect on the microbial community composition than on Alpha diversity. The direct effect of precipitation on community composition was positive, whereas its direct effect on Alpha diversity was negative (Figure 7a). Additionally, increased precipitation was not conducive to maintaining a neutral soil environment. Physicochemical soil factors did not significantly impact the community characteristics of nirS-type denitrifiers. However, temperature (Tem) and pH had negative effects on Alpha diversity to a certain extent, while TN also exerted negative impacts on community composition. In contrast, the effect of TC on microbial community composition was positive. Furthermore, Tem, TN, and TC may serve as crucial predictive factors for Alpha diversity and community composition of nirS-type denitrifiers under precipitation changes in the alpine wetland of Qinghai Lake (Figure 7b).

### 3.5. Process of Community Assembly of nirS-Type Denitrifiers under Precipitation Changes

Deterministic and stochastic processes jointly drive the community assembly of nirS-type denitrifiers. The calculation of βNTI values for community assembly under different precipitation treatments shows that deterministic processes dominate (|βNTI| > 2) at this regional scale (Figure 7c). Further calculation of RCbray distinguishes the relative impacts of dispersal limitation, drift, homogeneous dispersal, and selection on community dynamics (Figure 7d). Results indicate that, in the community assembly of nirS-type denitrifiers in Qinghai Lake wetlands, the relative influence of deterministic and stochastic processes varies under different precipitation treatments. Heterogeneous selection predominantly shapes the communities in different treatments, with drift playing a minor role in community assembly. Heterogeneous selection accounts for 100% in groups JA, Wck, and ZB, while drift influences community assembly under JB and ZA treatments, accounting for 33.3% and 66.6%, respectively.

## 4. Discussion

### 4.1. Precipitation Changes Had Little Effect on Alpha Diversity of nirS-Type Denitrifiers

Microbial communities heavily depend on water for mobility and matrix diffusion, and thus species diversity typically responds to changes in precipitation [35]. For instance, Zhou et al. [36] conducted a meta-analysis examining the influencing factors of soil microbial diversity and function. Their findings suggest that variations in precipitation can impact soil microbial diversity by altering soil pH values, both regionally and globally. Yang et al. [37] discovered that changes in precipitation indirectly influence microbial diversity by altering soil water content and pH. Meanwhile, Liang et al. [38] studied the characteristics of nirS and nirK communities in paddy soils of eastern China and concluded that climate factors, namely temperature and precipitation, along with pH value, are often crucial determinants of the diversity of nirS-type denitrifiers. Wei et al. [39] also suggested that pH was a critical variable in predicting alterations in the diversity of nirS-type denitrifiers. However, in the present study, precipitation changes across different gradients did not significantly impact the community diversity of nirS-type denitrifiers. Furthermore, their diversity was not significantly influenced by environmental soil factors, which contrasts with the findings of prior research. Nonetheless, Jiang et al. [40] investigated the impact of environmental variation and spatial distance on the denitrifying community of wetland soil and found no significant association between environmental factors and the abundance or diversity of nirS-type denitrifiers. This aligns with the findings of the present study. Furthermore, the pH of the alpine wetland in Qinghai Lake, under the influence of precipitation treatment, ranged from 6.20 to 6.71, showing narrow variation and exceeding the pH threshold of nirS-type denitrifiers, which is 4.7 [41]. Zuo et al. [42] also examined the comparative relationship between plant–soil microbial diversity under precipitation variations and demonstrated that precipitation changes primarily altered soil water content while exerting minimal influence on other soil properties. This could potentially account for the insignificant variations observed in the diversity index.

The richness of nirS-type denitrifiers increased in the alpine wetland under precipitation variations. Notably, the sensitivity of denitrifying microorganism richness was more pronounced in response to a 25% reduction in rainfall and a 50% increase in rainfall, contradicting our initial hypothesis. Nevertheless, our findings align with the research outcomes reported by Li et al. [43]. Their meta-analysis examined the impact of multiple global change factors on soil microbial richness, diversity, and functional gene abundance. Similarly, they observed that long-term precipitation changes facilitate an increase in soil microbial richness. Additionally, both the Shannon index and the Simpson index demonstrated a generally decreasing trend as precipitation increased. This contradicted our hypothesis that an increase in precipitation would positively correlate with the species richness and diversity of nirS-type denitrifiers. One plausible explanation is that the soil moisture exceeded a certain threshold, leading to osmotic stress and subsequently reducing diversity, as suggested by Kieft et al. [44]. Alternatively, the soil pH being less than 7, which is below the optimal pH environment for denitrifying bacteria, could have inhibited the denitrification process [45,46]. Qiu et al. [47] also discovered that the Shannon index of nirS-type denitrifiers had a direct negative impact on N_2_O emissions, suggesting that increased precipitation in the alpine wetland of Qinghai Lake may further exacerbate nitrous oxide emissions.

### 4.2. Precipitation Changes Significantly Affected the Interaction Network, Community Structure, and Functional Groups of nirS-Type Denitrifiers

Due to the crucial role of species interactions, encompassing both positive and negative as well as direct and indirect associations in ecosystem functions [48], the alterations in network complexity and the stability of microbial communities have garnered ongoing attention [49]. Consistent with Hypothesis 2, the present study found that the close association between microorganisms increased under most precipitation gradients. This may be attributed to the resistance and resilience exhibited by denitrifying bacteria in response to environmental stress [50]. However, a 25% increase in rainfall led to a decrease in the interrelationships among denitrifying microorganisms. This change may be influenced by alterations in soil nutrient content [51]. The dominant microflora at the phylum level in the alpine wetland of Qinghai Lake for nirS-type denitrifiers was Proteobacteria, which is consistent with previous research findings [6,38]. However, the dominant microflora at the genus level identified in this study differed from those reported in previous research. Specifically, the dominant bacterial genera identified in this study included *Rhodanobacter*, *Ideonella*, *Defluviicoccus*, and *Bradyrhizobium*. Wang et al. [52] examined the nir-type rhizosphere denitrifying bacterial community in constructed wetlands and discovered that *Sulfurifustis*, *Steroidobacter*, and *Pseudomonas* were the primary denitrifiers encoded by nirS at the genus level. Zhang et al. [53] studied the characteristics of nirS-type denitrifiers in Qinghai Lake wetlands under various vegetation types and found that *Azospirillum*, *Thiothrix*, *Rhodanobacter*, and *Bradyrhizobium* were the dominant bacterial genera. It is possible that the distinct wetland environments resulting from the diversity of wetland types influenced the microbial community composition at the genus level [54].

Changes in precipitation can affect soil water availability, influence plant community composition and productivity, and subsequently alter the abundance and composition of soil microorganisms, either directly or indirectly [55]. Previous studies have demonstrated that, with decreasing precipitation, the relative abundance of Proteobacteria exhibits an increasing trend [56]. Consistent with these findings, the present study observed a similar response trend in the relative abundance of Proteobacteria to precipitation changes, although no statistically significant difference was observed. Numerous studies have established a significant correlation between nirS-type denitrifiers and soil water content [57,58]. Similarly, the dominant bacterial genera associated with nirS-type denitrifiers in Qinghai Lake wetlands exhibited a pronounced response to precipitation changes, aligning with previous research findings. Specifically, *Rhodanobacter*, Ideonella, *Defluviicoccus*, and *Bradyrhizobium* demonstrated diverse responses to precipitation. *Rhodanobacter*, *Ideonella*, and *Bradyrhizobium* are aerobic bacteria, and their relative abundances were inconsistent with Hypothesis 3. The relative abundances of *Rhodanobacter* and *Ideonella* decreased gradually with increasing precipitation. The relative abundance of *Bradyrhizobium* increased significantly when precipitation increased by 25% but did not exhibit significant changes when precipitation increased by 50%. It is possible that insufficient precipitation may have had an adverse impact on microbial activity [59], whereas excessive precipitation may have reduced oxygen levels, thereby hindering the growth and reproduction of aerobic bacteria. *Defluviicoccus* is an anaerobic bacterium, and its relative abundance is positively correlated with precipitation, which aligns with Hypothesis 3. Furthermore, changes in precipitation significantly altered the relative abundances of the aerobic chemoheterotrophy, chemoheterotrophy, nitrogen fixation, ureolysis, and xylanolysis functional groups. This contradicts the conclusion reached by Zhang et al. [53]. However, Na et al. [60] found that precipitation changes disrupted the ecological functions of soil microbial communities, thus providing support for this study. The reverse localization of the five functional groups corresponding to the dominant microflora of nirS-type denitrifiers at the genus level revealed that they all belonged to Proteobacteria. This finding further verifies the crucial role of Proteobacteria in the nitrogen cycle process. It suggests that any changes in this bacterial group could have a significant impact on soil nutrients and potentially even the overall elemental cycle process [61].

## 5. Conclusions

This study thoroughly examined the dynamic responses of nirS-type denitrifiers to precipitation variations in the alpine wetland of Qinghai Lake. The findings revealed that precipitation changes had a profound impact on the community structure of nirS-type denitrifiers at the genus level, altering the relative abundance of critical functional groups. It is noteworthy that a 50% increase in rainfall shifted the community assembly process of denitrifying microbes from deterministic to stochastic. A 25% decrease in rainfall significantly elevated the relative abundance of three critical functional groups: chemoheterotrophic, aerobic chemoheterotrophic, and nitrogen fixation. Additionally, the reduction in rainfall resulted in an increase in the Shannon and Simpson indices. Proteobacteria were the dominant players within the denitrifiers. As precipitation increased, the complexity of microbial interactions decreased. Most importantly, precipitation has emerged as the most critical factor influencing the nirS-type denitrifiers, and an increase in precipitation may further elevate emissions of nitrous oxide in the alpine wetland ecosystem. This study offers valuable insights into how precipitation changes regulate denitrification processes in the alpine wetland ecosystem, providing a foundation for further research in this area.

## Data Availability

The raw data have been uploaded to NCBI, and its BioProject is PRJNA1041646.

## Supplementary Materials

The following supporting information can be downloaded at: https://www.mdpi.com/article/10.3390/biology13080596/s1.
