# Supplementary figures and images for: Response Characteristics and Community Assembly Mechanisms of nirS-Type Denitrifiers in the Alpine Wetland under Simulated Precipitation Conditions
Source: Biology (Basel). 2024 Aug 7;13(8):596. doi: 10.3390/biology13080596 (PMC11351468; doi:10.3390/biology13080596)

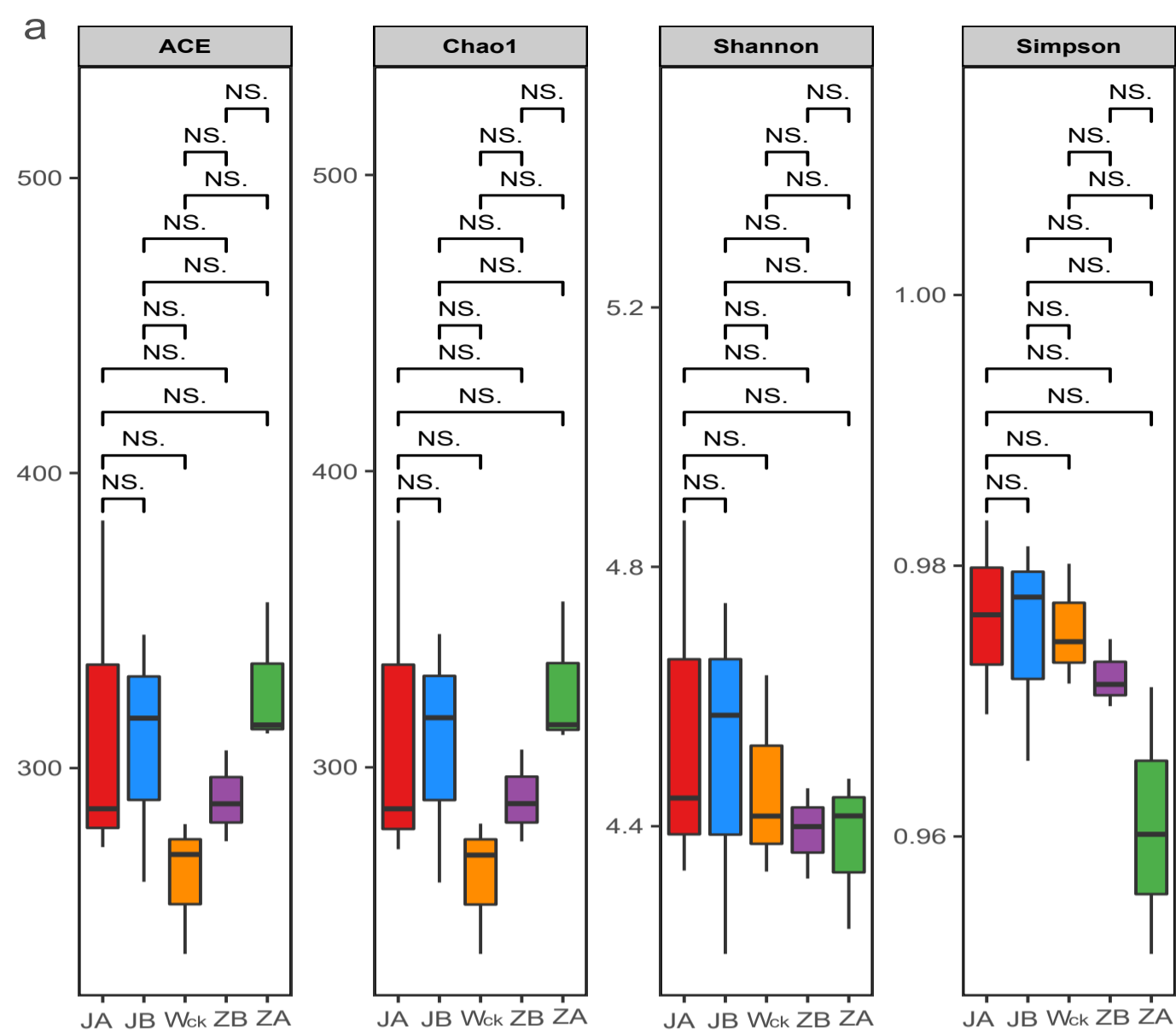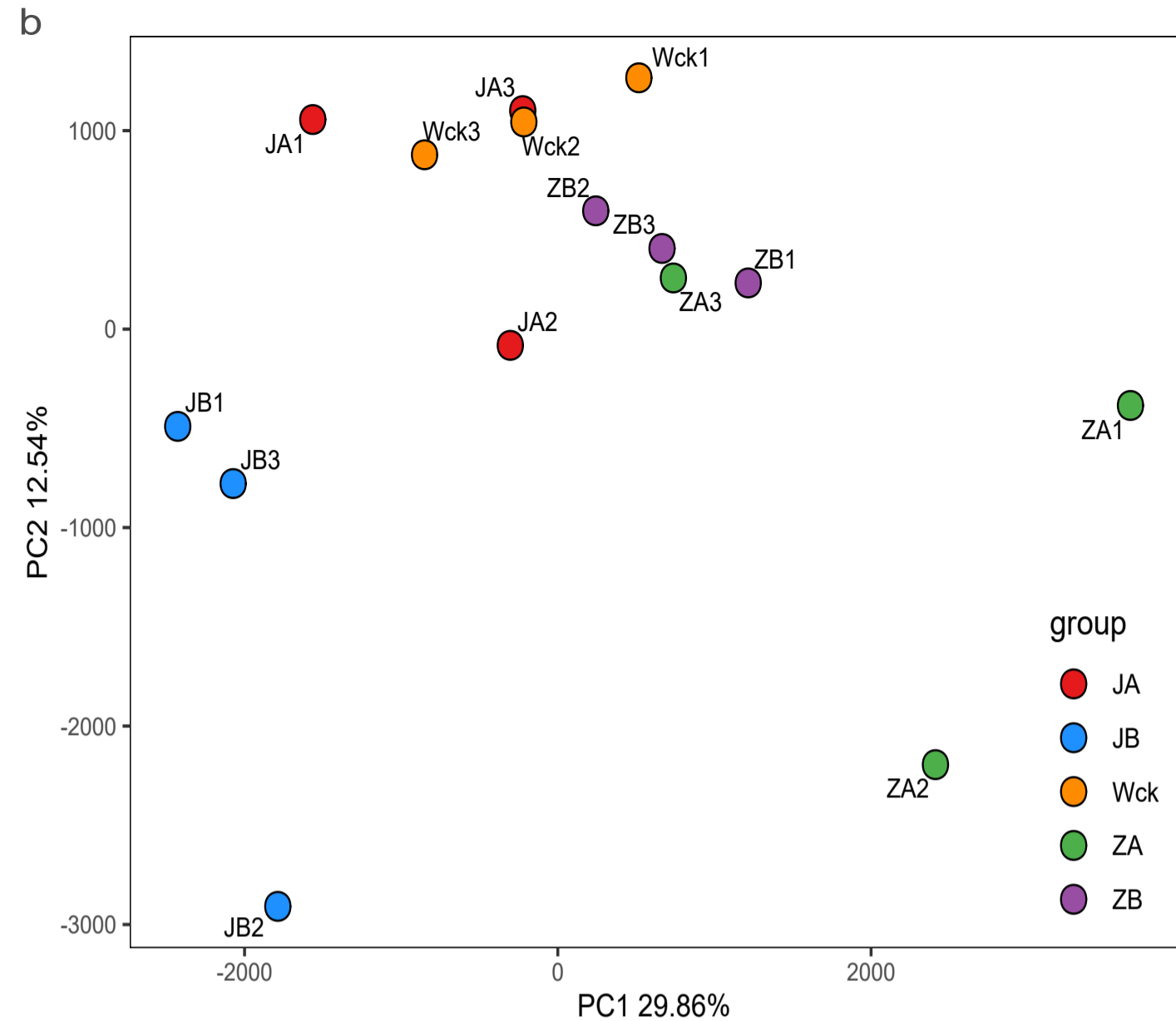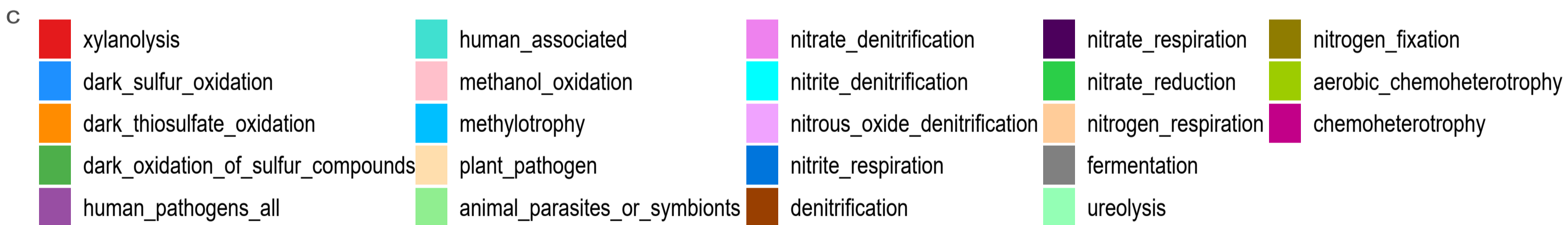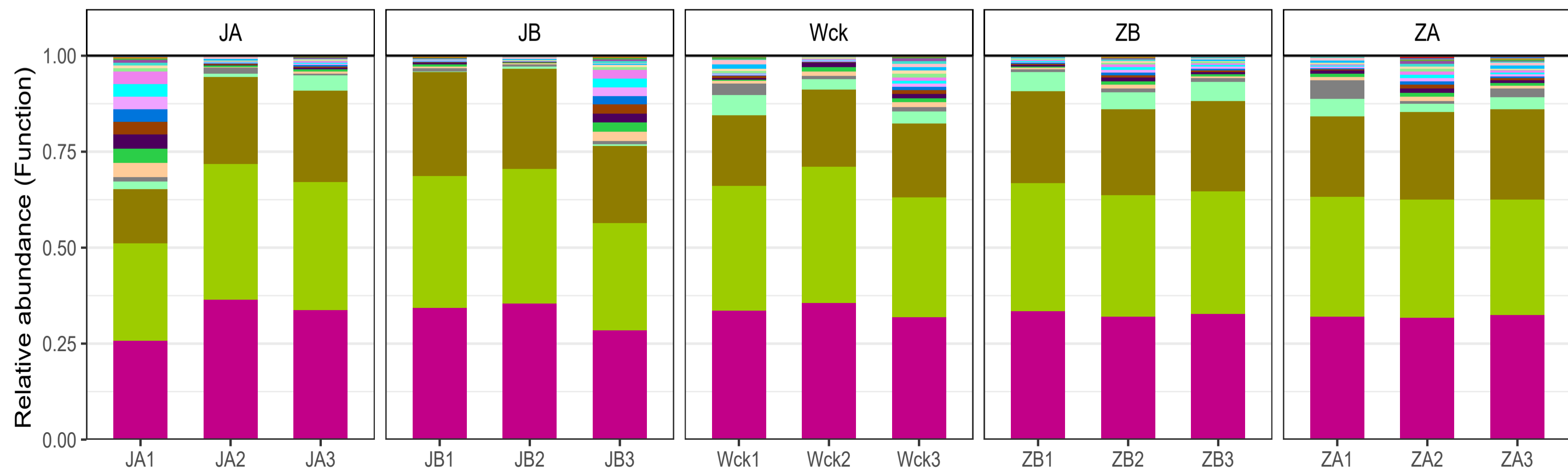

Supplement: Supplementary file 1 [file biology-13-00596-s001.zip › biology-3111598-supplementary.pdf]
